# A149 SAFETY AND EFFICACY OF ENDOSCOPIC SUBMUCOSAL DISSECTION FOR ESOPHAGOGASTRIC NEOPLASMS IN A CANADIAN SETTING

**DOI:** 10.1093/jcag/gwad061.149

**Published:** 2024-02-14

**Authors:** A AlDarwish, R Bechara

**Affiliations:** Western University, London, ON, Canada; Queen's University, Kingston, ON, Canada

## Abstract

**Background:**

Endoscopic submucosal dissection (ESD) is a technique that has been developed in Japan and is increasingly being adopted by western countries for treatment of superficial gastrointestinal neoplasms.

**Aims:**

In this study, we aim to present the safety and efficacy of ESD for esophageal and gastric neoplasms in a Canadian setting, given the limited data regarding the outcomes of ESD in North America

**Methods:**

Data of 100 patients with superficial upper GI neoplasms (esophageal and gastric) who underwent ESD between 2016 and 2022 in Kingston Health Sciences Centre, a tertiary hospital in Kingston, Ontario, were retrospectively reviewed. Demographics and lesion characteristics, ESD technique, and outcomes in terms of efficacy and safety were analyzed.

**Results:**

100 patients were included in the study. 67% of the lesions were esophageal. The median diameter was 4.6cm and median area was 11.94cm^2^. Outcomes were favorable with technical success 98%, en bloc resection 96%, R0 resection 89% and curative resection 80%. Upstage in pathology from index biopsy was seen in 42% of the lesions. Adverse events were infrequently encountered (8%) and included delayed bleeding, aspiration, and pain.

**Conclusions:**

ESD is a safe and effective modality for accurately diagnosing and treating early and superficial esophagogastric neoplasms when performed by trained endoscopists. Further research is required, however, to increase the adoption of this technique across Canada.

Procedural Details

**Funding Agencies:**

None

**Table:** 

Successful completion	98 (98%)
En bloc resection	96 (96%)
R0 resection	89 (89%)
Curative resection	80 (80%)
Procedure time (median)	86 mins (5 – 295)
Efficiency (median)	6.10min/cm2 (1.58 – 41.20)
Adverse events Aspiration Early bleeding Delayed bleeding Perforation Others	8 (8%) 1 (1%) 0 5 (5%) 0 2 (2%)

